# Use of the out-of-hours emergency dental service at two south-east London hospitals

**DOI:** 10.1186/1472-6831-9-19

**Published:** 2009-07-25

**Authors:** Rupert Austin, Kate Jones, Desmond Wright, Nora Donaldson, Jennifer E Gallagher

**Affiliations:** 1 Department of Oral Health Services Research and Dental Public Health, Kings College London Dental Institute, Caldecot Road, London, SE5 9RW, UK; 2 NHS Southwark Primary Care Trust, Mabel Goldwin House, 49 Grange Walk, London, SE1 3DY, UK; 3 NHS Lewisham Primary Care Trust, Cantilever House, Eltham Road, Lee, London, SE12 8RN, UK; 4 Unit of Biostatistics, King's College London Dental Institute, Caldecot Road, London, SE5 9RW, UK; 5 NHS Lambeth Primary Care Trust, 1 Lower Marsh, Waterloo, London, SE1 7NT, UK

## Abstract

**Background:**

Prior to the introduction of the 2006 NHS dental contract in England and Wales, general dental practitioners (GDPs) were responsible for the provision of out-of-hours (OOH) emergency dental services (EDS); however there was great national variation in service provision. Under the contractual arrangements introduced 1^st ^April 2006, local commissioning agencies became formally responsible for the provision of out-of-hours emergency dental services. This study aimed to examine patients' use of an out-of-hours emergency dental service and to determine whether the introduction of the 2006 national NHS dental contract had resulted in a change in service use, with a view to informing future planning and commissioning of care.

**Methods:**

A questionnaire was administered to people attending the out-of-hours emergency dental service at two inner city London hospitals over two time periods; four weeks before and six months after the introduction of the dental contract in April 2006. The questionnaire explored: reasons for attending; dental registration status and attendance; method of access; knowledge and use of NHS Direct; satisfaction with the service; future preferences for access and use of out-of-hours dental services. Data were compared to determine any impact of the new contract on how and why people accessed the emergency dental service.

**Results:**

The response rate was 73% of attendees with 981 respondents for the first time period and 546 for the second. There were no significant differences between the two time periods in the gender, age, ethnic distribution or main language of service users accessing the service. Overall, the main dental problem was toothache (72%) and the main reason for choosing this service was due to the inability to access another emergency dental service (42%). Significantly fewer service users attended the out-of-hours emergency dental service during the second period because they could not get an appointment with their own dentist (p = 0.002 from 28% to 20%) and significantly more service users in the second period felt the emergency dental service was easier to get to than their own dentist (P = 0.003 from 8% to 14%). Service users found out about the service from multiple sources, of which family and friends were the most common source (30%). In the second period fewer service users were obtaining information about the service from dental receptionists (P = 0.002 from 14% to 9%) and increased use of NHS Direct for a dental problem was reported (P = 0.002 from 16% to 22%) along with more service users being referred to the service by NHS Direct (P = 0.02 from 19% to 24%). The most common preference for future emergency dental care was face-to-face with a dentist (79%).

**Conclusion:**

This study has provided an insight into how and why people use an out-of-hours emergency dental service and has helped to guide future commissioning of these services. Overall, the service was being used in much the same way both before and after the 2006 dental contract. Significantly more use was being made of NHS Direct after April 2006; however, informal information networks such as friends and family remain an important source of information about accessing emergency dental services.

## Background

On 1st April 2006 new NHS dental contracts and new patient charges for NHS dental services were introduced to England and Wales. As part of this contractual change, primary care trusts (PCTs) in England became formally responsible for out-of-hours dental services [1]. There was media and public concern that the new arrangements might have resulted in dentists moving away from the provision of NHS dental services to the independent sector thereby reducing availability of NHS dental care [2]. Concern was also expressed that the new patient charging system could mean increased charges for people who opt for occasional rather than routine care. The resulting effect could be an increased demand and change in patterns of use of out-of-hours dental services.

Qualitative research on how and why people access emergency services suggests that advice and reassurance may be just as important as pain relief and that many attendees at a walk in emergency dental service would be happy with advice and a reliable appointment once surgeries re-opened [3,4]. Research in medical emergency departments highlights difficulty in accessing GPs as the major reason why people with primary care problems attend accident and emergency departments [5]. A survey of nationwide out-of-hours emergency dental service arrangements in the UK reported wide geographical variation in the provision of services, whilst local studies have reported relative dissatisfaction with walk-in arrangements [6,7], and improved access following the introduction of telephone triage [8-10].

The inner-city London boroughs of Lambeth, Southwark and Lewisham have had two established emergency dental services that were set up during the 1990s. These walk-in services were provided from King's College Hospital and Guy's Hospital. King's operated a two surgery system on weekday evenings and weekend and Bank Holiday mornings whereas the Guy's service only operated on weekends and bank holidays. Both services were funded by the local primary care trusts and staffed by local dentists. Most people self-referred; however, referrals were also made from various sources, including NHS Direct and local accident and emergency services. Both registered and non-registered NHS dental people used the service.

Information on how or why people access out-of-hours emergency dental services was required locally in Lambeth, Southwark and Lewisham to inform local commissioning under the new out-of-hours service arrangements. The study was also designed to provide a measure of the impact of the 2006 NHS dental contract on people's use of out-of-hours emergency dental services.

The aim of the study was to determine patterns of service use of the Lambeth, Southwark and Lewisham out-of-hours emergency dental service and to determine whether the introduction of the 2006 dental contract resulted in a change of patterns of attendance, characteristics of attendees and attitudes relating to attendance. This would inform future planning and commissioning of care.

## Methods

The study was designed as a two-stage cross-sectional questionnaire based survey. A questionnaire was administered during two time periods (March 2006 and October 2006) to all adults seeking dental care at the out-of-hours emergency dental services provided within Lambeth, Southwark and Lewisham. The questionnaire was developed using published research on how and why people access OOH EDS [6,10], and past local unpublished research into the characteristics of patient attending the emergency dental services in South East London. The questionnaire was piloted on 20 people prior to the start of the study. The principal inclusion criteria were all adult attendees who consented to participate in the study and who saw a dentist during their visit. Principal exclusion criteria were violent/aggressive/abusive attendees and children (17 and younger). Ethical approval was granted from The Central Office for Research Ethics Committees (COREC) and Research Governance approval was granted from King's College Hospital and Guy's and St Thomas' NHS Foundation Trusts and from Southwark PCT on behalf of Lambeth, Southwark and Lewisham PCT's.

**Figure 1:**
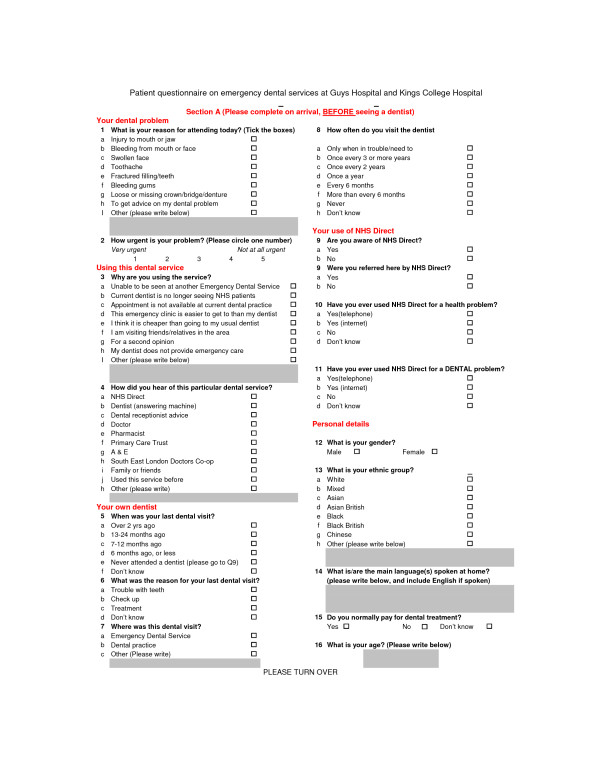
**Section A of questionnaire administered to service users attending an out-of-hours emergency service**.

**Figure 2:**
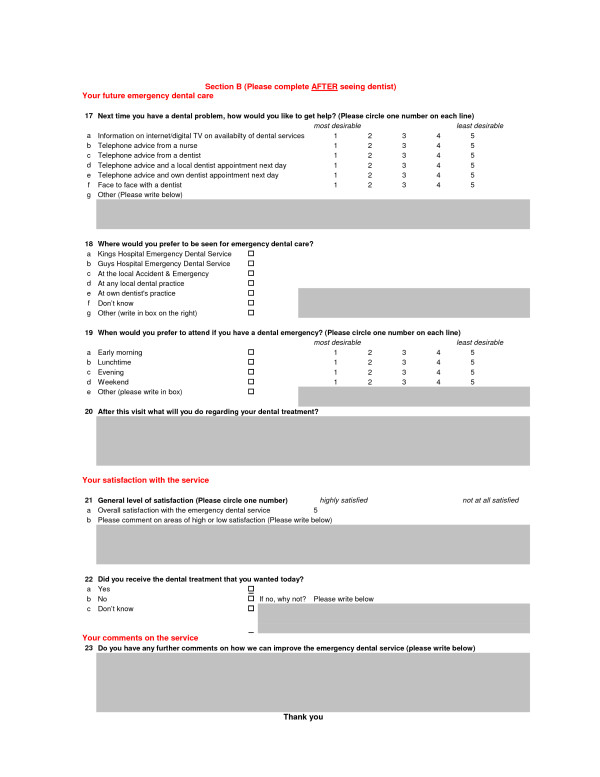
**Section B of questionnaire administered to service users attending an out-of-hours emergency service**.

In order to show equivalence of proportions of between 5% and 10%, with a maximum acceptable difference of 7%, an effective sample of 514 subjects was required for each time period. It was envisaged that the number of people recruited was going to be three to four times as large in King's in relation to Guy's due to the difference in the number of service users between each site. A questionnaire was administered to service users at the out-of-hours emergency service, on both hospital sites, by an administrator recruited and trained specifically for this task (Figures 1, 2).

The administrator invited patients to take part and asked them to complete the questionnaire in the waiting room. The themes of the questionnaire included:

◦ Section A (Figure 1)

▪ Reasons for attending the EDS

▪ Current dental registration status, and attendance

▪ How people accessed the service

▪ Knowledge of NHS Direct in accessing the EDS service, and satisfaction with accessing the service in this way

◦ Section B (Figure 2)

▪ Satisfaction with the service that they received on the day

▪ Preference of location and how people want to access future OOH care.

Section A of the questionnaire was completed before, and Section B after, seeing the dentist (Figures 1, 2). Questionnaires were collected in a labelled box at reception on each EDS site, supervised by the administrator and collected by the chief investigator. Demographic details were obtained from the Patient Information Management System. Data were analysed using SPSS for Windows software using parametric and non-parametric methods as appropriate to the corresponding response distributions. Initially, univariate comparisons of the two samples (over the two time periods) were undertaken using t-tests (or Mann-Whitney test) for continuous variables or chi-square tests (Pearson or chi-square test for trend) for categorical variables. Multiple regression was used to assess the differences in these outcomes between the two time periods, after adjusting for the effect of other covariates and possible interactions. The demographics-by-period interaction effects on those variables assessing change of attendance or attitude patterns were also explored.

## Results

In total, 981 questionnaires were completed in the first time period (of which 73% were at King's) and 546 questionnaires were completed in the second (of which 79% were at King's). In line with ethics committee approval, patients who declined to participate in the study were not required to give reasons for this and therefore no data were collected on refusals. Overall during the two time periods a total of 2092 patients attended the service, giving an overall response rate of 75% (Table 1).

**Table 1:** Number of service users attending an out-of-hours emergency dental service over two time periods

	**March 2006**		**October 2006**		**Total**	
	*N*	%	N	%	N	%
King's	718	73	432	79	1150	75
Guy's	263	27	114	21	377	25

Total	981		546		1527	

There were no differences in gender, age or ethnicity of service users between the two time periods of the study (Table 2). Respondents who had used the service before were compared with those who had not and no differences by demographic details were found. However, there were differences in ethnicity across the sites with Guy's having 75% (n = 283) White and 18% (n = 68) Black service users, compared with 53% (n = 610) White and 33% (n = 380) Black service users at King's.

**Table 2:** Gender, ethnicity, age and payment status of service users attending an out-of-hours emergency dental service over two time periods

		**March 2006**		**October 2006**		**Total**		**P-values**
		*N*	%	N	%	N	%	
Gender	male	468	51	255	49	723	50	0.38
	female	445	49	267	51	712	50	
Ethnicity	White	520	57	306	58	826	57	0.79
	Black	276	30	149	28	425	29	
	Others	123	13	70	13	193	13	
Age	18–64 years	857	97	491	98	1348	97	
	65+ years	30	3	11	2	41	3	
Normally pay for dental treatment?	yes	475	52	267	52	742	52	0.66
	no	376	41	206	40	582	41	
	don't know	58	6	39	8	97	7	

*Note*: Question has multiple responses therefore total ≠ 100%

### Nature of dental problem

Over both time periods, toothache was the main reason for attending the service (72%). There were no significant differences between the two time periods for the main reasons for attending (Table 3). In total, 90% of service users perceived their problem to be either 'urgent' or 'very urgent'.

**Table 3:** Reasons for attending amongst service users attending an out-of-hours emergency dental service over two time periods: Nature of dental problem

	**March 2006**		**October 2006**		**Total**		**P-Values**
	*N*	%	N	%	N	%	
Toothache	660	73	370	72	1030	72	0.62
Swollen face	147	16	103	20	250	18	0.08
Fractured filling/teeth	151	17	93	18	244	17	0.52
Loose or missing crown/bridge	76	8	52	10	128	9	0.29
To get advice on dental problem	64	7	37	7	101	7	0.94
Bleeding gums	49	5	34	7	83	6	0.36
Injury to mouth or jaw	38	4	19	4	57	4	0.63
Bleeding from mouth or face	20	2	21	4	41	3	0.04
Other reason	5	1	2	0	7	0	0.99

*Note*: Question has multiple responses therefore total ≠ 100%

### Reason for choice of service

When asked why they were using this particular service, 42% reported that they were unable to be seen at another emergency dental service and 27% claimed that their dentist did not provide emergency dental care. 7% of respondents claimed that their dentist was no longer seeing NHS patients. There were significant differences for these reasons between the two time periods (Table 4). Whilst 28% of service users reported that an appointment was not available at their dentist in the first time period, this was lower at 20% in the second (P = 0.002) and in the second time period a significantly higher proportion of service users reported that the EDS was easier to get to than their own dentist (P = 0.003).

**Table 4:** Reasons for attending an out-of-hours emergency dental service over two time periods: Reason for choice of service

	**March 2006**		**October 2006**		**Total**		**P-Values**
	*N*	%	N	%	N	%	
Unable to be seen at other Emergency Dental Service	303	41	170	43	473	42	0.6
My dentist doesn't provide emergency care	195	26	108	27	303	27	0.8
Appointment not available at my dentist	210	28	80	20	290	25	0.002
Easier to get to than my dentist	61	8	55	14	116	10	0.003
My dentist no longer seeing NHS patients	51	7	25	6	76	7	0.68
Cheaper than going to my dentist	12	2	7	2	19	2	0.87
Visiting friends/relatives in area	13	2	12	3	25	2	0.17
For second opinion	19	3	8	2	27	2	0.55
For other reason	10	1	10	3	20	2	0.16

*Note*: Question has multiple responses therefore total ≠ 100%

### How service users were directed to the service

The chief source of information about the service was from friends and family followed by NHS Direct. In the second time period, a significantly lower proportion had heard about the service from a dental receptionist (P = 0.002) (Table 5).

**Table 5:** How service users were directed to an out-of-hours emergency dental service over two time periods

	**March 2006**		**October 2006**		**Total**		**P-Values**
	*N*	%	N	%	N	%	
Family or friends	246	29	159	31	405	30	0.62
NHS Direct	188	22	127	24	315	23	0.39
Have used this service before	131	16	78	15	209	15	0.77
Dental receptionist	119	14	45	9	164	12	0.002
A & E	78	9	42	8	120	9	0.44
Dentist's answering machine	54	6	22	4	76	6	0.09
Doctor	28	3	16	3	44	3	0.79
Pharmacist	8	1	3	1	11	1	0.45
Primary care trust	10	1	7	1	17	1	0.8
South East London Doctors Co-op	12	1	8	2	20	1	0.87

*Note*: Question has multiple responses therefore total ≠ 100%

### NHS Direct

In response to questioning about the awareness and use of NHS Direct, overall 35% of service users had used NHS Direct for advice about a health problem and 18% for advice about a dental problem. Although no differences were detected between the two time periods in terms of awareness, there was an increase in referrals attributed to NHS Direct (P = 0.02) and increased use of NHS Direct for dental problems, in the second time period (P = 0.002) (Table 6).

**Table 6:** Use of NHS Direct amongst service users attending an out-of-hours emergency dental service over two time periods

		**March 2006**		**October 2006**		**Total**		**P-values**
		*N*	%	N	%	N	%	
Aware of NHS Direct	yes	571	64	340	67	911	65	0.27
Referred by NHS Direct	yes	147	19	109	24	256	21	0.02
Ever used NHS Direct for health problem?	yes – telephone and/or internet	290	35	171	36	461	35	0.76
	no	546	65	310	64	856	65	
Ever used NHS Direct for dental problem?	yes – telephone and/or internet	142	16	115	22	257	18	0.002
	no	771	84	398	78	1169	82	

*Note*: Question has multiple responses therefore total ≠ 100%

### Last dental visit

Overall the majority of service users reported visiting their dentist within the previous twenty-four months. In the first time period 80% reported that their last dental visit was at a dental practice whereas this figure was lower at 75% in the second; however, this difference was non significant (p = 0.08) (Table 7). Almost half of respondents (48%) reported attending a dentist less than every two years, with only 4% attending a dentist more than every six months (Table 8).

**Table 7:** Last dental visit amongst service users attending an out-of-hours emergency dental service over two time periods

		**March 2006**		**October 2006**		**Total**		**P-Values**
		N	%	N	%	N	%	
Last dental visit	Over 2 years ago	218	25	135	29	353	27	0.26
	13–24 months ago	103	12	48	10	151	11	
	7–12 months ago	139	16	76	16	215	16	
	6 months ago or less	400	47	205	44	605	46	
	Never attended a dentist	20	2	14	3	34	2	
	Trouble with teeth	401	43	213	43	612	43	
Reason	Check up	262	28	141	29	403	28	0.88
	Treatment	241	26	126	26	367	26	
	Emergency dental service	103	12	71	16	174	13	
Location	Dental practice	679	80	342	75	1021	79	0.08
	Other	63	8	43	9	106	8	

*Note*: Question has multiple responses therefore total ≠ 100%

**Table 8:** Frequency of dental visits amongst service users attending an out-of-hours emergency dental service over two time periods

	**March 2006**		**October 2006**		**Total**		**P-Values**
	*N*	%	N	%	N	%	
Every 3 or more years/only when need to/never	432	49	238	50	670	48	0.37
Every 2 years	51	6	33	7	84	6	
Once a year	138	16	85	18	223	16	
Every 6 months	221	25	103	22	324	24	
More often than every 6 months	41	5	20	4	61	4	

*Note*: Question has multiple responses therefore total ≠ 100%

### Future emergency dental care

With regard to how service users would like help for a future dental problem, the preferred choice was face-to-face with a dentist followed by telephone advice with an appointment the next day. The least preferred option was using the internet or digital TV. There were significantly higher proportions of service users preferring face-to-face contact with a dentist and a significantly lower proportion preferring phone advice (p = 0.02) followed by a next day appointment at their own dentist in the second time period(P = 0.03) (Table 9).

**Table 9:** Future preferences for accessing emergency dental care amongst service users attending an out-of-hours emergency dental service over two time Periods

		**March 2006**		**October 2006**		**Total**		**P-values**
		N	%	N	%	N	%	
How would like to get help – face to face with dentist	1: Most Desirable	380	78	270	82	650	79	0.02
	2	33	7	27	8	60	7	
	3	29	6	18	5	47	6	
	4	11	2	1	0	12	1	
	5: Least desirable	37	8	14	4	51	6	
How would like to get help – phone advice with own dentist next day appt	1: Most Desirable	348	66	168	55	516	62	0.03
	2	98	18	70	23	168	20	
	3	44	8	43	14	87	10	
	4	12	2	10	3	22	3	
	5: Least desirable	29	5	15	5	44	5	
How would like to get help – phone advice with local dentist next day appt	1: Most Desirable	188	44	89	34	277	40	0.13
	2	117	27	85	32	202	29	
	3	69	16	51	19	120	17	
	4	21	5	20	8	41	6	
	5: Least desirable	34	8	17	6	51	7	
How would like to get help – phone advice from dentist	1: Most Desirable	188	41	86	32	274	37	0.1
	2	109	23	62	23	171	23	
	3	71	15	69	25	140	19	
	4	51	11	33	12	84	11	
	5: Least desirable	45	10	22	8	67	9	
How would like to get help – phone advice from nurse	1: Most Desirable	98	23	36	14	134	20	0.06
	2	92	22	46	18	138	20	
	3	89	21	81	31	170	25	
	4	72	17	57	22	129	19	
	5: Least desirable	77	18	38	15	115	17	
How would like to get help – internet/digital TV	1: Most Desirable	77	18	26	10	103	15	0.06
	2	54	13	33	13	87	13	
	3	75	17	52	20	127	18	
	4	42	10	35	13	77	11	
	5: Least desirable	183	42	115	44	298	43	

*Note*: Question has multiple responses therefore total ≠ 100%

With regard to what time of the day people would like to attend for emergency dental care, the preferred choice both before and after the introduction of the new contract was early morning. The responses in order of preference were: early morning (75%), evening (61%), weekends (53%) and lunchtime (29%). Significantly greater proportions of service users in the second survey (after the introduction of the 2006 dental contract) indicated a preference to attend in the evenings (p = 0.0001) and at a weekend (p = 0.02) (Table 10).

**Table 10:** Future preferences for timing of emergency dental care amongst service users attending an out-of-hours emergency dental service over two time periods

		**March 2006**		**October 2006**		**Total**		**P-values**
		N	%	N	%	N	%	
Prefer to attend early morning	1: Most desirable	447	77	211	72	658	75	0.21
	2	47	8	27	9	74	8	
	3	28	5	18	6	46	5	
	4	14	2	10	3	24	3	
	5: Least desirable	46	8	26	9	72	8	
Prefer to attend evening	1: Most desirable	235	56	197	69	432	61	0.0001
	2	55	13	28	10	83	12	
	3	64	15	35	12	99	14	
	4	27	6	12	4	39	5	
	5: Least desirable	42	10	15	5	57	8	
Prefer to attend weekend	1: Most desirable	165	49	126	59	291	53	0.02
	2	49	15	25	12	74	13	
	3	34	10	26	12	60	11	
	4	29	9	18	8	47	9	
	5: Least desirable	57	17	20	9	77	14	
Prefer to attend lunchtime	1: Most desirable	87	27	62	32	149	29	0.5
	2	109	34	41	21	150	29	
	3	46	14	39	20	85	17	
	4	27	8	10	5	37	7	
	5: Least desirable	53	16	41	21	94	18	

*Note*: Question has multiple responses therefore total ≠ 100%

### Satisfaction with the service

Overall 95% of service users were satisfied with the out-of-hours service; however, issues were raised around unmet expectations of an emergency dental service and a lack of information about treatment and charges available. There were no significant differences between the two time periods.

### Improving the service

The service users were asked whether they had any further comments on how the service could be improved. Improving staff numbers and extending opening hours were the most common suggestions. Other areas for improvement included shorter waiting times, better facilities, better treatment options and improved information on the website.

## Discussion

The introduction of a new national NHS dental contract in 1992 provoked a number of studies during the 1990s about attendance patterns [11-15], which highlighted the increasing demand for emergency dental services. Subsequently, research focussed on ways of managing demand and improving access [8,9] including a move to use of telephone triage. However, by 2000 research highlighted wide variation in the provision of services and suggested that increased use of services such as NHS Direct may help to reduce inequity of access [16]. Further qualitative research examined expectations of and satisfaction with emergency dental services in order to further understand some of the reasons how and why people access out-of-hours emergency dental services [3,4,6,7,10].

### Service users numbers and personal characteristics

This study had a good response rate of over 70% and the differing response sizes (with King's providing 75% of the service users) are explained by the differing size of service provision at the two geographic sites. The differences in geographic location could explain the varying ethnicity of service users, with unpublished hospital attendance data showing that around 72% of service users at Guy's were people from outside the immediate boroughs such as commuters, reflecting its proximity to a mainline railway station, whereas 69% of service users at King's are from the local boroughs, whose population is characterised by a socially deprived, ethnically diverse and mobile population.

### Nature of dental problem

Overall, the majority of service users were attending with the same pattern of problems (toothache, swollen face and fractured tooth/filling) over the two time periods, suggesting that six months into the new contract there was little change in the reasons for attending the service. This pattern of service use was similar to findings in previous studies of attendance at an emergency dental service [4].

### Patterns of service use

There was little evidence of change in the major influences determining the patterns of service use; however, the results of this study suggest that after the introduction of the 2006 dental contract fewer service users were likely to report that their own dentist would not see them or that they had been referred on by a receptionist. The popular perception at the time was of difficulty in accessing a dentist, heightened by negative media coverage. This may have dissuaded emergency patients from attempting to get an appointment directly with the dentist resulting in them going direct to the EDS or via NHS direct, which made significantly more referrals. Alternatively, primary dental care may have been more able to absorb emergency patients, resulting in less referral on from providers to the EDS. Either way this fits with the overall trend of reducing primary dental care uptake following the introduction of the new contract.

### NHS Direct

After the introduction of the 2006 dental contract more service users were being referred to the service by NHS Direct and more service users were using NHS Direct for advice about a dental problem. This suggests increasing awareness and use of NHS Direct for dental problems and is in line with national policy around future commissioning of out-of-hours care using telephone triage systems to prioritise according to need. The increasing use of NHS Direct may also reflect either real or perceived problems that service users had with accessing a general dental service. Studies of general medical practice have shown that use of NHS Direct may limit demand for out-of-hours care [17,18], and the experience of NHS Direct itself is that a high percentage of callers can be successfully managed by telephone advice followed by an appointment with a local dentist during normal working hours.

### Limitations and benefits of the study

As already noted, the study was carried out against a period of major policy change in the provision of primary dental care during which widespread negative media coverage focussed on the difficulties in accessing an NHS dentist for routine dental care [2]. The interval between studies was six months, which although long enough for service evaluation and to inform local commissioning, may not have been sufficiently long enough to evaluate the effect of a policy change as service users may not have been aware of a change in primary dental care provision; this may be the reason for the similar patterns of service use before and after the introduction of the new dental contract.

The study design was a two-stage cross-sectional survey. Therefore the respondents constitute different service users at different time points, without a control group. This is a potential source of bias, however there were no significant differences found in the personal characteristics of the respondents at the two time periods, which strengthens the internal validity of the study.

Recognising the above limitations and the fact that this was a study of an emergency dental service provided within the locality of a dental teaching hospital in inner-city London, the findings will therefore be useful for any other organisation developing new models of out-of-hours care for emergency dental services by providing valuable information on how and why people access these services.

### Implications for commissioning, policy and research

There is increasing public expectation around provision of care outside normal working hours and in line with these trends, the Department of Health is reforming the pattern of delivery of care for it to fit better around people's busy lives [19]. However, if a significant proportion of the population is accessing an out-of-hours emergency service for convenience due to the relative ease of access of this type of service, then there may need to be a method of triaging cases to allow those who need urgent care to access it appropriately [20]. The Department of Health recommendations for commissioning out-of-hours services include the use of dental triage systems in either open or closed door systems in order to prioritise according to need [1].

Following this study, local commissioning organisations have made major changes in the provision of emergency dental services, which are now accessed via telephone triage. Patients needing urgent care receive a same day appointment with less urgent cases referred directly from telephone triage to dental practices. Dental contracts have been revised to reflect these changes, which include extending opening hours and also allowed for the creation of open access slots during the working day. These appointment slots during normal working hours are kept free for emergency patients who either contact the practice directly or who are referred via the telephone triage service. As patient satisfaction with the direct access service was generally positive, further research is needed on evaluating outcomes of new models of emergency care, following changes in service provision.

This study has also highlighted the importance of friends and family in providing knowledge about out-of-hours emergency dental care, despite the increasing awareness and use of NHS Direct. In light of the importance of informal networks of knowledge, commissioners should therefore consider strategies for communicating health service information to the public on accessing these important services.

## Conclusion

This study has provided an insight into how and why people use an out-of-hours emergency dental service and has helped to guide commissioning of emergency dental services in south-east London. Despite a major change in the contractual arrangements for the provision of primary dental care, users continued to access emergency care in much the same way except for some minor changes in line with wider trends, such as more use of NHS Direct and a preference for future emergency dental care at the evenings and weekends. Informal information networks such as friends and family remain an important source of information about accessing emergency dental services. In line with these trends, NHS organisations will need to consider how to commission services in future, an important element of which should be a strategy to communicate information about the service to the public.

## Competing interests

DW, KJ and JG provided Dental Public Health specialist support and advice to Lambeth, Southwark and Lewisham PCTs who commission and fund out-of-hours dental services.

## Authors' contributions

DW was involved in design of the study, fieldwork, analysis of data and contributed to the write up of the paper. ND was involved in design, analysis and contributed to the write up of the paper. JG was involved in the design, analysis and contributed to the write up of the paper. RA was involved in the analysis and contributed to the write up of the paper. KJ was involved in the design and contributed to the write up of the paper. All authors approved the final manuscript.

## Pre-publication history

The pre-publication history for this paper can be accessed here:


